# Barriers to Accessing Oncology Services for Effective Cancer Care in the Public Health Institutions in Limpopo Province, South Africa: A Qualitative Study

**DOI:** 10.3390/nursrep13030084

**Published:** 2023-07-12

**Authors:** Neo Jacqueline Ramutumbu, Dorah Ursula Ramathuba, Maria Sonto Maputle

**Affiliations:** Department of Advanced Nursing Science, Faulty of Health Sciences, Main Campus, University of Venda, Thohoyandou 0950, South Africa

**Keywords:** barriers, cancer care, co-ordination, medical devices, mortality, support services

## Abstract

It is estimated that by 2030, 24 million people worldwide will develop cancer, and 13 million will die annually, with 75% of deaths in low- and middle-income countries. The management and effective control of care have not been fully achieved due to a lack of material and human resources exacerbated by poor governance and co-ordination of the services. The study aimed to explore barriers to accessing oncology services for effective cancer care in the public health institutions in Limpopo province. The study was conducted in the five district hospitals in Limpopo province. A qualitative exploratory descriptive and contextual approach was used to collect data that employed focus group discussions amongst healthcare professionals in different disciplines. Non-probability purposive sampling was used to sample participants from various sections contributing to oncology care. Five focus group discussions were conducted at the selected hospitals. The data were analysed using the eight steps of Tesch’s method. The findings revealed that Limpopo province has a shortage of high-technology medical equipment, poor coordination, and a lack of oncological and allied expertise. Governments should ensure that patients receive the care required as stated in the constitution to navigate cancer care pathways to improve patient health outcomes, particularly in rural areas where care is fragmented and poorly financed. Recommendations to support oncology patients involve psychosocial work and palliative care of the multidisciplinary teams to be put forward. The identified barriers regarding oncology care may contribute to changing the departments’ outlook and effective functioning by including interdisciplinary oncology teams at all levels of care.

## 1. Introduction

Cancer is an expanding health problem in Africa, and due to the cost of care and the absence of facilities, cancer mortality rates are expanding in Africa. Cancer death rates in Africa are projected to exceed the global average by 30% in the next 20 years [1]. Cancers can be prevented by avoiding risk factors and implementing evidence-based prevention strategies. The cancer burden can also be reduced through early cancer detection and appropriate treatment and care of patients who develop cancer [2]. Southern Africa is a high-incidence area with a pattern of risk factors due to lifestyle changes, including urbanisation, adopting Western lifestyle habits, and increasing tobacco and alcohol consumption. Hamdi et al. [3] indicated that cancer has received low priority for healthcare services in sub-Saharan Africa. Furthermore, the authors concluded that several disparities in cancer diagnosis and screening between the different African regions contribute to the morbidity and mortality rates across regions. Dalton et al. [4] indicated that high-income countries have successfully implemented strategies for patient navigation systems along the cancer care continuum, strategies which are lacking in low-income countries, resulting in ineffective cancer care.

Kruk et al. [5] indicated that approximately 8 million people in low middle-income countries (LMICs) die from causes related to ineffective health care; of these, 5 million people who used the health system received poor-quality health care. The deaths attributable to receiving poor-quality health care constitute 58% of all amenable mortalities in LMICs other than the non-utilisation of services. Ineffective and poor-quality health services are holding back progress in improving health in countries with low-income levels. Ineffective quality health care is predominantly occurring in poor rural communities due to the socio-economic status of those communities. Government subsidies are often insufficient and lacking. Coupled with poor healthcare knowledge in rural communities, patients access inefficient or inadequate care due to a lack of resources, diagnostic machinery, good medications, and specialised skills [6]. Poorly trained staff delay referrals to more specialised services. The inefficient co-ordination and fragmented pathways in cancer care at all stages, including prevention, symptom awareness, diagnosis, treatment, and post-treatment care, make cancer hard to manage, thus increasing mortality rates [6]. Tetteh et al. [7] highlighted similar challenges to access to oncology services, such as sociocultural barriers and weak healthcare systems limiting patients’ navigation services in LMICs, as most patients enter the health system at the primary care level. In Limpopo province, cancer care services are ineffective because they are not well co-ordinated, and shortages of clinical doctors at the district level to assess, diagnose, refer, and provide treatment are not prompt, leading to poor cancer care outcomes. South Africa reported that neoplasms caused 41,799 deaths, accounting for 9.1% of all adult deaths in 2015. The infrastructure and capacity to treat adults with cancer are variable throughout the country. Many tertiary specialist units are grossly understaffed and poorly equipped, and thus are unable to provide holistic care for the majority of patients [8].

Effective cancer care refers to implementing comprehensive and proven measures to address the burden of cancer actively. These measures start from prevention through health education campaigns, screening services for early detection, accurate diagnosis, and staging, providing prompt, effective treatment through access to the right combination of surgery, radiotherapy, chemotherapy or supportive care, and regular specialist follow-up. Palliative care and rehabilitation are crucial in the cancer trajectory, including psychosocial support and critical communication between clinical teams, patients, and carers. A cancer service can prevent, cure, control, and manage palliative symptoms.

The ineffective cancer care provided at public hospitals is a cause for concern in decreasing the quality of life of the population in Limpopo province. Inadequate cancer care and co-ordination are major contributing causes of the morbidities and mortalities in the region. The study may highlight the need for the health department to prioritise cancer services and budgets to provide practical, comprehensive cancer care.

## 2. Problem Statement

Cancer is the second leading cause of death globally, and impacts patients negatively. It causes significant physical, emotional, and financial strain on patients, their families, the community, and the health system [2]. The South African health system does not have universal medical coverage for every citizen, and most patients are uninsured and cannot afford specialised cancer care. Mukwevho [9] reported the death of a woman who succumbed to cervical cancer while waiting to be transferred due to a long list of patients awaiting an appointment for a consultation with an oncologist at Limpopo Provincial Hospital. A deficit of oncologists is a structural issue within the public health system in the province, as only one oncologist located at the provincial health facility is tasked with treating cancer patients from all 43 regional and district hospitals. The community and regional hospitals do not have oncology services. Oncology patients are admitted to the general wards with other chronic conditions. Patients wait months to be consulted by the only oncologist, creating a massive backlog and a lack of access to cancer care. Shortages of skilled practitioners at the district and community level make assessment, early diagnosis, referral, and provision of treatment and care impossible, thus rendering cancer care and co-ordination ineffective.

There is increasing concern about the equity problem, specifically regarding the accessibility of specialist treatment and care. The government of the day declared universal access to health care as a fundamental principle, and the national insurance health plan was instituted so that everyone should receive adequate and equitable care. However, national health insurance seems to be failing marginalised communities, as some of the health services selected as pilot centres failed to provide access to specialised treatments and medicines. The system fails patients in accessing quality comprehensive oncology services due to a lack of clinical and non-clinical staff with different competencies and specialities; this results in substandard care because the patients visit the facilities several times without proper consultations from skilled practitioners, and by the time the patient is referred, the disease has progressed. At rural public facilities, patients do not receive accurate screening, early detection, and appropriate treatment. Mukwevho [9] indicated that Dr Aron Motsoaledi announced during his tenure as Minister of Health to triple the number of doctors to 3600 in preparation for national health insurance in 2014; however, there is still a challenge with placing interns in public hospitals due to financial constraints. The inequity in oncology care services does not assure survivorship in rural health settings. In this regard, many cancer patients fall ill and die from disease progression and metastases without proper medical attention.

Access to cancer care is limited at primary healthcare clinics and public hospitals. Patients are reluctant to consult or be referred for further management at far away institutions, which results in cancers not being diagnosed and managed early, due to the inaccessibility of cancer services within their reach. The appointment system is prolonged, as the only oncologist in the province cannot consult all the referrals. At times with the same appointment, the patient may return without being consulted due to the long queues or the oncologist having other commitments, resulting in a new booking having to be made for the patient. The appointment system is poorly co-ordinated and controlled, as patients queue again to retrieve files and wait at the outpatient department before being seen. The bookings do not have timeslots for patients; when it is time to knock off, the patients’ transport must return patients to their respective district hospitals because they are seen on an outpatient basis. Malan [10] reiterated that incomplete treatment, lack of resources, and lack of screening were also highlighted as barriers related to health facilities. In South Africa, there is a lack of a proper model of care for oncology services in public hospitals, especially in rural areas. Based on the above, the researchers sought to explore the barriers to accessing oncology services for effective cancer care in the public health institutions in Limpopo province.

## 3. Research Design and Methods

A qualitative exploratory descriptive and contextual research design was utilised to explore and describe the barriers to accessing oncology services for effective cancer care in the public health institutions in Limpopo province [11]. The qualitative exploratory design research approach assists researchers in understanding the natural context of the participants as they detail how they experience the phenomena under study; the researchers can capture meaningful characteristics related to real-life events as experienced and narrated. The participants could share their experiences regarding providing cancer care in the public hospitals of Limpopo province.

## 4. Research Setting

Limpopo province is the country’s northern most province, having international borders with Botswana, Mozambique, and Zimbabwe. Limpopo province has five districts; the study was undertaken in the five public hospitals in the district municipalities. The province has challenges of lacking specialised human resources, medicine stock shortages, and extended patient waiting times. It is a poor rural province; a majority of the population is uneducated, unemployed, uninsured, and reliant on government pension grants. The health system provides free essential services through the district health support system, with community health workers assisting with caring for patients with chronic diseases and HIV.

No specialised health service is provided. In public hospitals, sophisticated tests such as mammographies and computed tomography (CT) scans, for example, are carried out at provincial hospitals, and only by an appointment/booking system. Cancer care services require early and proper diagnosis and treatment. The Ministry of Health has also been providing specialist care by mobilising specialists from other provinces to come and reduce the backdrop of surgeries, and prioritise attention to paediatric cancers at a given time. The health system lacks specialists, as most doctors want to work in urban areas with better facilities and infrastructure. 

## 5. Population and Sampling

The target population was healthcare professionals providing oncology care in the five district hospitals for a year; those with less than 1 year of experience were excluded. A sample of doctors, professional nurses, social workers, and psychologists was purposively achieved. Sampling bias was prevented by including all doctors, psychologists, and social workers, as they were a small sample, unlike professional nurses who were in the majority. The participants were conveniently approached by the primary researcher, who explained the study’s purpose and methods. Participants gave verbal consent, and after that, the participants were contacted by the researcher and written consent was signed, and time and date arrangements were made for the focus group interviews.

## 6. Data Collection

After submitting the provincial approval letter, participants for the focus group interviews were invited through their supervisors and managers. Then, the invitation letters and information leaflets were sent to the relevant wards and support staff offices to recruit participants. Written consent was obtained prior to focus group commencement. The focus group discussions (FGDs) sample ranged from four to six members (professional nurses, oncology nurses, doctors, social workers, and psychologists). However, four of the five selected or sampled hospitals did not have a psychologist due to human resources factors. A conducive non-threatening environment was created where the seating was arranged in a semi-circle and made as informal as possible. The research assistant who moderated the session welcomed everyone, let each participant introduce themselves, and outlined the house rules, ensuring communication was open and frank, and no one overpowered the conversations. Furthermore, the participants consented to the recordings of the discussions. The opening question that directed the focus group discussions followed by the probing was *“What are the barriers to the effective provision and co-ordination of oncology services and care in your daily practice?*” A total of five focus group discussions were conducted at the selected hospitals, one per facility, and the discussions lasted for 45 to 60 min in the offices or boardrooms provided by the respective public hospitals. The data were gathered between April 2019 and July 2019. The focus group sessions were conducted by experienced interviewers and audio recorded. The focus group schedule contained straightforward, direct questions that probed issues regarding barriers to cancer care and early diagnosis, barriers to cancer care access and referral pathways, and suggestions for service improvements in the pathways to cancer diagnoses. FGDs were a suitable data collection method because the detailed and elaborated data generated through social interaction of a group are often deeper and richer than those obtained from one-to-one interviews.

## 7. Data Analysis

The primary author transcribed the focus group audio recordings, and all authors performed inductive thematic analysis, where preliminary codes and categories were identified through further discussions. Coding themes and sub-themes were constructed following Tesch’s steps [12]. The audio tape data collected and analysed are reported and discussed in this manuscript’s categories of themes and sub-themes; no additional data are available.

## 8. Trustworthiness

Trustworthiness was achieved through the model of [13,14]. The truth value, applicability, consistency, and neutrality were criteria used to determine the value of the findings. The truth value was achieved through member checking. Follow-up was carried out with focus group members to validate the data gathered, and field notes were written to ensure that the observations were captured. An audio recorder was used to record the focus group interviews. A stepwise replication procedure was used where transcripts were individually read and re-read, and categorised to build themes and sub-themes; applicability was achieved through purposive selection criteria that involved a multidisciplinary team of health professionals, and the communication techniques increased credibility through rephrasing, paraphrasing, repeating, and more probing. Consistency was achieved by all researchers to reach a consensus after discussing the themes and sub-themes. The availability of all transcripts and field notes for the confirmability audit achieved neutrality.

## 9. Ethical Consideration

Ethical approval to conduct the study was obtained from the University Research Ethics Committee (UREC), project registration SHS/18/PDC/25/0812. The chief executive officers of the health services of Limpopo province granted permission for the study to be conducted at the hospitals. Ethical principles regarding research participants, such as respect for persons, privacy, protection from harm, confidentiality, beneficence, and the right to self-determination, were applied to the study participants. The purpose and research processes were explained, and information leaflets were also provided. Participants who volunteered to participate after information giving were required to sign consent forms.

The participants’ anonymity and confidentiality were maintained throughout the research process through the use of numbers to identify participants and focus groups. The identities were only known by the researcher, in order to maintain client confidentiality. The participants were given due respect in their own fields of expertise; the purpose of the study was explained, and they were advised to be free to communicate their thoughts so they would not be judged. If they felt uncomfortable answering some questions, they would not be pressured, and could terminate their participation without penalty.

## 10. Findings

Three themes and sub-themes emerged from the findings, which are supported by verbatim quotations from participants. They are written in italics, and indicate the gender, age, and occupation of the participant. The results of the focus group discussions are presented in Table 1. The participants were identified by codes (i.e., D—doctor; SW—social worker; P/N—professional nurse), gender, and age. The focus groups were labelled as FGD#, and the groups were identified as A, B, C, D, and E.

## 11. Demographic Data

The participants in the study were a group of healthcare professionals consisting of medical doctors, professional nurses, oncology-trained nurses, social workers, and psychologists. Their ages ranged from 28 to 59 years, and their professional experiences ranged from 5 to 26 years.

## 12. Demographic Profile of the Participants in Focus Groups

The study participants were a heterogeneous group of healthcare professionals: 3 medical doctors, 12 professional nurses, 2 oncology-trained nurses, 4 social workers, and 3 psychologists. Their ages ranged from 28 to 59 years, and their professional experience ranged from 5 to 26 years. The focus group consisted of both genders.

## 13. Presentation of Findings

### 13.1. Theme 1: Shortage of Skilled Human Resources in Oncology Care

Cancer management requires various health professionals, and cancer disease makes people suffer physically, psychologically, emotionally, and spiritually. The multidisciplinary team needs to support all the spheres and care of cancer patients.

***Shortage and lack of skilled health professionals***

The participants expressed their concerns regarding the need for a skilled multidisciplinary team; however, the health system lacked skills in this category of health professionals.

Hereunder are the verbal quotes of what they had to say:

*“When patients are referred to the social services before being discharged or should come back for the service, they often are reluctant due to long queues as the social services are for every patient in the hospital. There are only one or two in the hospital at a time. Sometimes patients are referred to their community social worker and never keep the appointments.”*

[Female, 33 years, P/N]

*“We are just medical practitioners, but we refer patients, but sometimes you hit the wall; either the is a long list, or the oncologist is not available is having a busy schedule, and patients come back unattended……mmmh…. and some patients never return.”*

[Male, 37 years, D]

A participant in the same FGD#D shared the need for skills:

*“I have realised the need to be able to communicate to the cancer patients about their plight and psychosocial care needs, most important knowledge and education on cancer care to improve my relationship with them.”*

[Female, 30 years, SW]

The participant implied that there are fewer social workers to deal with patients’ needs when the patients are still in the hospital, as there is a long waiting list for the patients to see a social worker. Furthermore, social workers implied that they lack the knowledge and skills to satisfy the cancer patients’ psychosocial care and support needs. Rural patients lack support due to limited sub-specialist healthcare providers and local community support services.

***Overworked healthcare personnel***

The hospitals have a dire shortage of professionals, let alone specialised personnel such as oncologists, psychologists, and social workers, in addition to a shortage of facilities necessary for general patient care. Cancer care is physically and psychologically taxing for healthcare personnel.

A participant in FGD#B indicated the following:

*“It is so challenging to deal with all the patients available; at our hospital, there are no psychologists, as nurses, you have to make sure you do patient and family care and counselling.”*

[Female, 33 years, P/N]

Another participant in the same focus group indicated the following:

*“The nurses are so overworked that they fall sick in the wards and are getting sick and often booked for long sick leave, and as such, those remaining in force with the load of work cannot cope with.”*

[Female 57 years, P/N]

Another participant in the same focus group indicated the following:

*“As a social worker, I sometimes wish to follow up on some patients that I see, go to their home, and establish a relationship to be able to know of their social environment to make certain recommendations for financing and social support; there is no time and transport to do the tasks as we have to order transport from the pool and at least two days before when an emergency occurs, transport is prioritised for other services, sometimes there are meetings that were not planned, and you miss patients appointments that you have scheduled. It disappoints the patients and makes you inefficient and challenged”*

[Female, 30 years, SW].

The participants felt overwhelmed by the stressful working conditions they were experiencing while providing oncology care.

### 13.2. Theme 2: Poor Patient and Administrative Support in Cancer Services

Effective administration and adequate management of cancer patients make life easier for the patients. It decreases the patient’s anxiety, reassures the patient, and reduces patient uncertainties due to the cancer diagnosis.

***Delays in the diagnosis related to misinformation***

Delays in diagnosis and treatment services for cancer are associated with insufficient knowledge about cancer, limited or a lack of access to available services, and delays in seeking health care. Participants indicated a failure to seek early medical attention due to lack of information, reluctance, or the indigenous belief system.

The health workers in FGD#D expressed this as a challenge:

*“The patients are not coming to the hospital as they identify changes in their bodies, some are too busy to come to the hospital, and some are not able to identify the signs earlier.”*

[Female, 36 years, SW]

Another participant in FGD#D further explained the challenge:

*“In my community, patients rely mostly on the faith healers and the traditional healers before they can come to the hospital.”*

[Female, 39 years, P/N]

Participants implied that rural communities seek support from their traditional people, and use traditional remedies before consulting the hospitals. They are uncomfortable with the long queues and shortage of doctors, which delays early diagnosis and treatment. The health system should involve the patient’s family throughout the cancer care continuum so that they can act as a source of support for the patient to cope with the diagnosis.

***No reports on follow-up of the patients***

The communication among the specialists is often fragmented and seldom team-oriented, leading to communication gaps among them and their patients. Patients are shared between the primary and tertiary hospitals, and sometimes patients never bring reports to continue their care management to the referring hospital. As such, patients get lost within the system, with no follow-up appointments.

A participant in FGD#D indicated the following:

*“It is important for the reports to be sent back to the referring hospital with the advice on how to continue with the treatment of the patient, patients’ default, and you meet them after a long time in a devastating condition after months of defaulting.”*

[Female, 39 years, P/N]

It was evident from the participants’ discussion in this focus group that there is inadequate communication among the primary healthcare workers and specialists. The system will reduce defaulting treatment on the part of the patients if communication is adequately managed. Communication must be established and maintained among oncologists within and between allied and support services, in order to address communication gaps among them and the patients.

***No available staff to navigate patients to the treatment centres***

The need to navigate cancer patients is relevant; still, patients often go independently, due to staff shortages and hospital transfer policies. They struggle within the health system due to unusual settings at the other hospitals and the language barriers experienced in tertiary health settings.

Healthcare professional FGD#B spoke about the challenge of navigation:

*“We nurses are so short staffed, you find a patient who is supposed to go for their first-time consultations being transferred along with other patients who are going to other specialists, and all are allocated only one nurse, who knows nothing about the patient’s medical history.”*

[Female, 33 years, P/N]

Another participant in the same FGD#B indicated the following:

*“Patients get emotionally and physically affected being tossed between care providers.”*

[Female, 30 years, SW].

Patients must feel safe in the healthcare system, and must be navigated through their cancer journey. Navigating cancer is focused on enabling personalised cancer care for every patient. It provides a single, co-ordinated platform to deliver comprehensive cancer care for cancer patients. Patient navigation services are associated with improved access to timely diagnosis, treatment, and follow-up.

### 13.3. Theme 3: Poor Co-Ordination of Cancer Care Services

Cancer care co-ordination is critical to improving care quality and ensuring desirable health outcomes. Cancer patients require care that is holistic and comprehensive. There is a need to improve health care, the role of intra-sectorial collaboration in cancer treatment, and increase access to cancer treatment centres and services in rural hospitals.

***Loss of patient files and laboratory results***

The rural health system is still practising hospital-retained patient record systems. Manual capturing and storing of hard copies delays retrieving files and flow regarding patients’ medical needs and follow-up on the test results, such as for blood work and X-rays.

A health worker in FGD#B spoke of records getting lost:

*“Patient’s records are vital and create confusion if they are lost in the central file rooms. No one can be held responsible for the loss; by the time the patient finds the file, it is too late, the doctor or the health professional is gone, or the patient cannot wait for interaction, either due to transport or other social commitments beyond their control.”*

[Female, 54 years, P/N]

Another participant in FGD#C indicated the following:

*“The notes of the sickness and those of the social workers do not need to mix; as such, in her office, she summarises what she discusses with the patients as they always come with a new file.”*

[Female, 30 years, SW]

The participants are implying that the missing files contribute to prolonged waiting times for assistance. Thus, nurses and doctors cannot help patients or treat them immediately without their previous medical history. Proper filing of patients’ medical records ensures easy retrieval, and contributes to decreased patient waiting times at the hospital, as well as greater continuity of care.

***Lack of cancer policy guidelines/protocols***

The guidelines and policies are essential for patient safety in health care, preventing patient harm in healthcare facilities. It aims to prevent and reduce risks to patient safety.

A participant in FGD#C indicated the following:

*“When I see cancer patients, I always thought someone would do the rest. I was only doing my part, which I thought was only a part of what the patient needed, it never crossed my mind about cancer care and management policies.”*

[Female, 30 years, SW]

A participant in FGD#C indicated the following:

*“Once you see a patient and order what you should, you believe that the nurses and the matrons know what protocols are to be followed; we never make a follow-up, even if the patient is to be transferred, it is always up to them to arrange, and we trust that they have been doing this for ages.”*

[Male, 33 years, D]

The availability of cancer care policies in the hospitals would direct the cancer services to a more direct service that the cancer patients need that would be satisfactorily fulfilled, and could improve their quality of life.

***Inadequate and inefficient collaborative support in cancer care***

Collaboration in health care has improved patient outcomes, as expertise adds a specific value, creating a collective synergy to meet patients’ needs most effectively. However, some health professional teams may lack skills, and ignore patients’ needs.

A participant in FGD#B indicated the following:

*“I am very busy at the office, but since the beginning of the year, I have only seen only one patient with cancer, and it was for a social reason and need for financial aid only; I did not know what else to discuss with him about his illness, I felt so dumb could not Google up in his presence, I wish we need a little orientation on cancer and other essential hints on how to support and counsel them.”*

(Male, 32 years, SW).

He added the following:

*“I think we need to be oriented into the general needs of the cancer patients and what to assist them with as they visit our offices, maybe by workshops or constant meetings with the people directly involved in the cancer patient’s care.”*

(Male, 32 years, SW)

The participants agreed that there is a need for interprofessional collaboration in patient care to meet cancer patients’ psychosocial care and support needs. Interprofessional collaboration and communication are critical for quality and safety in healthcare delivery.

## 14. Discussion

The study sought to explore barriers to effective oncology care services. The findings provided evidence of a lack of skilled health professionals in oncology care services, creating poor cancer management and co-ordination. A cancer diagnosis is burdensome, as it affects the patient physically, psychologically, and financially. Therefore, a co-ordinated team of multidisciplinary health personnel is required to manage the patient holistically and comprehensively. The healthcare system has a shortage of multidisciplinary specialists required in oncology care. Patients referred from district hospitals do not always see oncologists for their cancer care during their visit, due to either workload or other work-related commitments of the only oncologist in the province.

de Vos et al. [14] indicated that allied health professionals compose 60% of the healthcare workforce, and despite this large number, laboratories nationwide are experiencing a shortage of qualified technologists. The lack of physicians in many specialities affects the quality of cancer care. The lack of oncologists in the public health services and the province creates a massive barrier to cancer care access. Patlak et al. [16] indicated that there are few specialised health personnel in African countries, such as oncologists and pathologists. The number of oncologists in Africa ranges from 0 to 1500; this is related to the high patient workload per physician. Many cancer patients may not even see a medical oncologist in consultation.

Another barrier contributing to the lack of skilled human resources is that cancer patients are seen by primary care physicians or general practitioners in community service rather than by oncologists or experienced medical practitioners in public healthcare services. South Africa’s specialist doctor shortage is most severe in rural public hospitals. Partnerships between universities and the private sector, as well as a scholarship for sub-speciality training, could be some initiatives that allow undergraduate students studying at public universities to spend some months in clinical training at private hospitals under the supervision of local hospital specialists to upskill themselves. Specialists can benefit by becoming accredited lecturers and by teaching students who could later become referring doctors. These sub-specialist categories can significantly impact rural healthcare settings in the early diagnosis of malignancies [17]. However, in South Africa, even the interns suffer placement issues after training due to a lack of finances by the health department to absorb them into the health workforce.

Oncology care also requires other sub-specialists for such fields as gastroenterology, surgery, dermatology, radiology, urology, gynaecology, haematology, pathology, pulmonology, internal or family medicine, in addition to other support staff such as social workers and psychologists. These sub-specialists are also lacking in complementing oncology care. The findings indicated no psychologists in most hospitals, and only one psychologist based at a regional hospital. The social workers believed they lacked the necessary competencies and skills to support oncology patients. Patlak et al. [16] also reported that the social worker shortage affects cancer care involvement, including patient assessment for grants, financial support, and counselling—and that they were helping patients cope with cancer-related depression and anxiety.

Furthermore, the theme revealed overworked healthcare personnel; this translates to poor or ineffective care, as patients’ needs are not provided as expected, and care is not holistic. Overwork leads to fatigue, and fatigue leads to a greater chance of mistakes. Excessive overtime negatively impacts patient care and increases the risk of ‘compassion fatigue,’ including desensitisation and loss of empathy [18]. Research also indicates that a heavy nursing workload adversely affects patient safety. Furthermore, it negatively affects job satisfaction and, as a result, contributes to high turnover and the nursing shortage.

The study participants revealed there was poor co-ordination of cancer care services due to rural hospitals lacking adequate technology and still using paper-based patient records. These contribute to the poor co-ordination of services due to records becoming misplaced or lost, resulting in delays in patient diagnosis or secondary care to services. A study conducted in the United Kingdom (UK) [19] reported that clinicians are challenged with making decisions about patient care without essential items of clinical information. Fifteen percent (n = 1161) of patients seen in the outpatient clinics had missing data; of those patients with missing clinical information, 32% experienced a delay or disruption to their care, 20% had a risk of harm, and in over half of the cases, the doctors relied on the patient for the information. Other issues that paper-based health records present include unclear handwriting, incomplete or inaccurate information, and inaccessibility of health records from various locations, exposing patients to medical mistakes because healthcare providers cannot draw connections between the current and past medical history. Burnett et al. [20] also indicated that resources such as fax referrals may get lost, and inflexible hospital booking systems and the unavailability of equipment or staff can result in missed or cancelled appointments.

Furthermore, differences in hospital systems, like tertiary hospitals compared to community hospitals, can leave patients disempowered, frustrated, and confused when experiencing difficulties in accessing healthcare services. Patients’ files can go missing during follow-up consultations, and new files are reopened with missing previous medical information. This is a barrier, as patients move two steps back instead of one step ahead in receiving adequate care. The authors are of the view that health systems should introduce electronic medical records to reduce missing clinical information. Most rural populations are illiterate and cannot comprehend medical language; electronic medical records may facilitate greater integration between primary and secondary care.

The participants in this study indicated that as much as they are not well-informed about cancer care, there is poor collaborative support in cancer care. The multidisciplinary teams work in silos, and others are not aware of the activities of other health professionals. No groups meet to discuss the co-ordination of patient care. The department should develop policies and guidelines on managing disease groups, referral pathways, and roles of other allied workers in supporting the medical team. Capacitating healthcare professionals is essential for patient care and support; it assists the professionals in reaching the patients in the sphere that they lack. It also provides insight into the ability of the professional to identify other deviant issues in the patient’s health to provide total care. Walton et al. [21] suggested that multidisciplinary team (MDT) meetings should be integral to delivering co-ordinated, collaborative care. MDT meetings allow different clinicians to discuss treatment options, offer alternatives to treatment paths that have been chosen, clarify the role, and update the team on new treatment protocols.

The study’s findings revealed delays in patient diagnoses. Patients travel long distances for a specialist machine and, at times, only to find that it is not working or there is no specialist on duty to operate the technological device because the person is on leave or committed. Furthermore, there are no follow-up reports on the patients, and the oncologist does not write back to the primary physician who referred the patient, which impedes adequate co-ordination and care. A lack of specialised skills is a challenge in the rural province. Cancer patients in South Africa are bearing the brunt of the disease and the health system. According to [22], South Africa faces a shortage of radiographers, ageing equipment, a significant oncologist crisis, a progressive decrease in clinical and radiation oncologists, and an overload of cancer patients.

Poor rural patients are escorted to the provincial hospital for a consultation, and one nurse accompanies three patients to different speciality clinics, which is frightening and frustrating for patients with poor language comprehension and cultural and personal barriers. The accompanying nurse is also not conversant with the patient’s medical history. Patients were previously accompanied by non-medical volunteers or caregivers, but the model also did not bear fruit in Limpopo due to financial constraints. Balasubramanian et al. [23] recommended integrating patient navigators into the care team to promote an optimal benefit for the client and improve co-ordination and communication between team members and caregivers. The navigators are a point of contact to mitigate system disparities and ensure care integration. Patient navigation is a novel approach to health service delivery that can improve care integration across disciplines, settings, levels of care, and sectors. Doucet et al. [24] also reported an increase in the proportion of patients who returned for follow-up after abnormal clinical breast examination findings after implementing the breast navigation program at Aga Khan University Hospital, Nairobi.

Patients cannot always comprehend what an oncologist may be implying or misunderstanding, so they cannot accurately report the facts to the primary healthcare provider. When communication is clear, co-ordination in the continuity of care will improve. Seeth [25] indicates that primary care practitioners (PCPs) require written communication about their patients’ diagnosis prognoses and care from the oncologists, especially when patients complete cancer treatment, as some continue going to primary care services for follow-ups.

## 15. Limitations

The limitation of the study was sample representativeness, as the healthcare professionals sample mainly constituted allied healthcare and professional nurses, with few medical doctors; therefore, their views may be over-represented compared to those of the doctors.

## 16. Recommendations

- Private hospitals and specialists should participate in the clinical training of undergraduate students to increase sub-specialities.
- To address the shortage of specialist doctors, the health fraternity and government should stop restrictions on the employment of foreign doctors and the limited training capacity or ratios coupled with the high requirements for specialist training.
- The government should provide economic incentives and academic status for working in rural health settings.
- The provincial government should budget for and increase high-technology equipment and resources to aid in early diagnosis and prompt treatment and referral where necessary.
- Treatment centres or mobile oncology clinics should be decentralised at regional hospitals in the districts to allow access to follow-up treatment.

## 17. Conclusions

The study identified shortages in human resources to provide comprehensive cancer care, poor patient administration and support, and poor co-ordination of cancer service. The strategies recommended by the participants indicate that human and system factors are required to improve patient satisfaction and health worker self-actualisation on seeing patients recuperate and experience a quality life. The strategies recommended by the participants indicate that a system to mitigate risks caused by human and system factors is required to improve patient satisfaction in Limpopo’s healthcare system. Overall, this study has provided a basis for future research and development of an oncology navigation system.

## Figures and Tables

**Table 1:** Themes and sub-themes.

Theme	Sub-Themes
Shortage of human resources in oncology care	Shortage and lack of skilled health professionals; overworked healthcare personnel
Poor patient administration and support in cancer services	Delays in diagnoses due to misinformation; no reports on follow-up of the patients; no available staff to navigate patients to the treatment centres
Poor co-ordination of cancer care services	Loss of patient files and laboratory results; lack of cancer policy guidelines/protocols; inadequate and inefficient collaborative support in cancer care

Source: Ramutumbu, N.J., 2021. “Support strategies for improvement of cancer services in the hospitals of Vhembe district, Limpopo Province.” PhD thesis, unpublished, University of Venda. [15].

## Data Availability

All data supporting this manuscript have been made available. All data transcripts coded and analysed during this study are included in this article.

## Public Involvement Statement

No public involvement in any aspect of this research.

## Guidelines and Standards Statement

This manuscript was drafted against the Standard for Reporting Qualitative Research (SRQR) [26] and the COREQ guidelines [27]. A complete list of reporting guidelines can be accessed via the equator network: https://www.equator-network.org/.
